# Mulberrin Alleviates Renal Ischemia–Reperfusion by Inhibiting Ferroptosis and Oxidative Stress Through Sirt3 Activation

**DOI:** 10.3390/biomedicines13112687

**Published:** 2025-10-31

**Authors:** Qiangmin Qiu, Zhan Chen, Wenbin Yang, Yujie Zhou, Nan Jiang, Jiahao Jiang, Dalin He, Yifan Lu, Bo Yu, Tao Qiu, Jiangqiao Zhou

**Affiliations:** 1 Department of Organ Transplantation, Renmin Hospital of Wuhan University, Wuhan 430060, China; 2015302180359@whu.edu.cn (Q.Q.); applepure@163.com (Z.C.); zhouyujie0514@163.com (Y.Z.); nanjiang0730@163.com (N.J.); 17607137591@163.com (J.J.); 17326375859@163.com (D.H.); yifanlu26@gmail.com (Y.L.); yubo1995@whu.edu.cn (B.Y.); 2 Department of Urology, Renmin Hospital of Wuhan University, Wuhan 430060, China; 3 Department of Emergency, Renmin Hospital of Wuhan University, Wuhan 430060, China; 18207135709@163.com

**Keywords:** renal ischemia–reperfusion injury, ferroptosis, oxidative stress, Sirtuin 3

## Abstract

**Background:** Renal ischemia–reperfusion (I/R) injury represents a principal etiologic factor in acute kidney injury (AKI), in which ferroptosis plays a critical role. Mulberrin (Mul), a prenylated flavonoid with antioxidative properties, has an as-yet undefined role in renal I/R injury. **Methods:** We established a mouse renal IRI model and an HK-2 H/R system. Renal function, histological injury, oxidative stress, ferroptosis markers, and mitochondrial function were assessed. The role of Sirtuin 3 (Sirt3) in Mul-mediated effects was further examined using siRNA knockdown in HK-2 cells. **Results:** The administration of Mul led to a marked improvement in renal function, lessened tubular injury, and reduced apoptosis in IRI mice. Mul also restored GSH levels, decreased MDA and Fe^2+^ accumulation, and normalized expression of ferroptosis-related proteins, thereby suppressing ferroptosis. In H/R-injured HK-2 cells, Mul restored mitochondrial membrane potential, increased ATP production, and reduced ROS accumulation. Mechanistically, Mul markedly upregulated Sirt3 expression, and silencing Sirt3 abolished its antioxidant and anti-ferroptosis effects, confirming the essential role of Sirt3 in Mul-mediated protection. **Conclusions:** Our findings underscore Mul’s therapeutic promise in acute kidney injury and provide a mechanistic foundation for interventions directed at the Sirt3–ferroptosis pathway to safeguard renal function.

## 1. Introduction

Acute kidney injury (AKI) denotes an abrupt impairment of kidney function, typically evidenced by an increase in serum creatinine and oliguria [1]. The condition occurs in approximately 10–15% of general inpatients and in as many as 50% of ICU admissions. Its clinical impact is considerable, with elevated risks of complications and death, and it predisposes survivors to chronic kidney disease and progression to end-stage renal disease [2]. In this context, delineating the principal etiologies of AKI is critical for targeted prevention and therapy. Renal ischemia–reperfusion injury (IRI) is a major cause of AKI, commonly arising during renal transplantation, cardiovascular surgery, and sepsis [3]. IRI induces tubular epithelial cell injury and death through oxidative stress, mitochondrial dysfunction, and inflammatory responses [4,5]. Despite advances in supportive care, no effective specific therapy for AKI exists, and renal replacement therapy remains the primary option. Therefore, it is imperative to explore novel strategies, and accumulating evidence suggests that natural medicines may provide promising therapeutic potential by targeting key mechanisms of AKI.

Ferroptosis represents a comparatively new form of regulated cell death whose molecular and morphological signatures differ from those of apoptosis, necrosis, and autophagy. The process is iron-dependent and driven by peroxidation of membrane phospholipids [6]. Hallmarks include excess cellular iron, reduced glutathione (GSH), functional suppression of glutathione peroxidase-4 (GPX4), and accumulation of lipid-derived ROS. On electron microscopy, affected cells exhibit diminished mitochondrial volume, increased membrane electron density, and disappearance of cristae [7]. A growing body of work demonstrates a pivotal contribution of ferroptosis to IRI [8,9]. Excessive iron and oxidative stress during IRI exacerbate tubular epithelial cell injury, while inhibition of ferroptosis with specific inhibitors, such as deferoxamine or liproxstatin-1, has been shown to alleviate renal dysfunction in experimental models [10,11]. These findings highlight ferroptosis as a key pathological mechanism in renal IRI and suggest that targeting ferroptosis represents a promising therapeutic strategy for AKI.

Sirtuin 3 (Sirt3), the principal NAD^+^-driven deacetylase within mitochondria, orchestrates energy metabolism, limits oxidative stress through antioxidant defenses, and sustains mitochondrial homeostasis and functional stability [12,13]. Recent studies have further revealed that Sirt3 acts as a critical regulator of ferroptosis, not only via the Sirt3/p53 axis but also by deacetylating and stabilizing GPX4, a key enzyme that prevents lipid peroxidation [14,15]. Moreover, the Sirt3/HO-1 axis has been implicated in ferroptosis regulation, highlighting the interplay between mitochondrial redox balance and iron metabolism [16]. Evidence indicates that Sirt3 affords renoprotection in ischemia–reperfusion settings by curbing oxidative damage and maintaining mitochondrial integrity. However, the mechanistic basis is not fully defined, and it is presently unclear whether these benefits are mediated by the inhibition of ferroptosis [17]. Therefore, this study aims to explore whether Sirt3 confers renoprotection against I/R injury through its antioxidative and anti-ferroptosis mechanisms.

Mounting evidence supports traditional Chinese medicine as a promising therapeutic strategy for AKI [18]. Mulberrin (Mul), also known as Kuwanon C, is a prenylated flavonoid derived from Morus alba [19,20,21]. Prior studies indicate that Mul mitigates spinal cord injury by dampening neuroinflammation, limiting oxidative stress, and reducing neuronal loss in both animal models and cell-based systems [22]. Moreover, Mul has been reported to modulate the Nrf2 signaling pathway to alleviate oxidative stress and inflammation, thereby ameliorating liver fibrosis [23]. Despite ample evidence supporting the antioxidant properties of Mul, its role in renal IRI remains undefined. In this study, we set out to determine whether Mul exerts protective effects in renal IRI and to elucidate the molecular pathways through which such protection might occur.

This study shows that Mul protects against renal IRI in both animal and cell models. Mechanistically, Mul exerted its effects by activating Sirt3 and inhibiting ferroptosis. Our results support Mul as a potential therapeutic candidate for preventing ferroptosis and mitigating renal damage following IRI.

## 2. Methods

### 2.1. Experimental Animals

Male C57BL/6 mice (20–25 g, 4 weeks of age) were purchased from the Laboratory Animal Center of Wuhan University School of Medicine. Animals were randomly allocated to four cohorts (*n* = 5 each): sham surgery, IRI, IRI + Mul at 20 mg/kg, and IRI + Mul at 40 mg/kg. The renal I/R model followed a published protocol [17]. After right nephrectomy, the left renal pedicle was clamped with a non-traumatic microvascular clip for 30 min, then released to permit reperfusion; the left kidney was harvested 24 h after reperfusion for analysis. Sham mice underwent nephrectomy without pedicle clamping. Mul was administered intraperitoneally once daily for three consecutive days prior to surgery at either 20 or 40 mg/kg, and DMSO served as the vehicle control. All animal experiments adhered to the institutional guidelines of the Renmin Hospital of Wuhan University (Wuhan, China) and received approval from the Experimental Animal Ethics Committee (Approval No. WDRM20250309B).

### 2.2. Cell Culture and Transfection

HK-2 human renal tubular epithelial cells (obtained from the China Center for Type Culture Collection) were maintained in complete growth medium within a humidified incubator at 37 °C with 5% CO_2_. To model ischemia–reperfusion in vitro, we implemented a hypoxia/reoxygenation sequence: cells were placed in DMEM lacking glucose and serum under 1% O_2_ (37 °C, 5% CO_2_) for twelve hours, then returned to complete medium for six hours of reoxygenation [24]. Mul was applied twenty-four hours before the H/R challenge at 10, 20, 40, 80, or 160 μM, and DMSO was used as the vehicle control.

For gene silencing, siRNA targeting Sirt3 (si-Sirt3) and a negative control siRNA (si-NC) were synthesized by Sangon Biotech Co., Ltd. (Shanghai, China). Transfections were carried out with Lipofectamine 2000 (Invitrogen, Shanghai, China) in accordance with the supplier’s protocol.

The siRNA sequences were: sense, 5′-AGAAGAGATGCGGGACCTTG-3′; antisense, 5′-GGTCCATCAAGCCTAGAGCAG-3′.

### 2.3. Renal Function Tests

Mice were anesthetized with 2.5% Avertin and bled via retro-orbital venipuncture. Samples were allowed to clot and centrifuged to obtain serum. Creatinine (Cr) and blood urea nitrogen (BUN) were measured using manufacturer-approved assays (C011-2-1 and C013-2-1; Nanjing Jiancheng Bioengineering Institute, China).

### 2.4. Immunohistochemistry (IHC)

Paraffin-embedded renal sections were processed using an UltraSensitive™ SP IHC Kit (Fuzhou Maixin Biotech Co., Ltd., Fuzhou, China). Sections underwent microwave-assisted antigen retrieval in citrate buffer, were blocked with 3% BSA for 30 min, then incubated with anti-Sirt3 (1:200, Cell Signaling, #2627S), anti-KIM-1 (1:100, Cell Signaling, #14971S), an-ti-GPX4 (1:100, Cell Signaling, # 52455), or anti-ACSL4 (1:100, Cell Signaling, # 38493). After PBS washes, sections were incubated with HRP-conjugated secondary antibodies in 1% BSA. DAB was developed to optimal intensity for 1–3 min, followed by thorough rinsing, hematoxylin counterstain, bluing, dehydration, clearing, and mounting [17]. Isotype and no-primary controls were included and images from random cortical fields were acquired at 200× magnification.

### 2.5. Hematoxylin and Eosin (H&E) Staining

Whole renal were fixed in 4% paraformaldehyde, processed through ascending ethanol series, embedded in paraffin blocks, and microtomed into 4-μm slices. After xylene-based deparaffinization and aqueous rehydration, sections received hematoxylin staining and an eosin counterstain. Slides were subsequently dehydrated, cleared, and mounted using a neutral resin medium [25]. Histopathological features were evaluated under a light microscope, and representative images from the renal cortex were captured at 200×.

### 2.6. TdT-Mediated dUTP Nick-End Labeling (TUNEL)

TUNEL staining was carried out on kidney sections with the Beyotime C1090 kit (Beyotime, Shanghai, China) per the manufacturer’s protocol. After permeabilization and labeling, slides were washed, DAPI-counterstained, and imaged on an Olympus IX51 (Olympus Corporation, Tokyo, Japan) fluorescence microscope [17]. The fraction of apoptotic cells was calculated from ≥5 randomly selected fields as TUNEL-positive/total nuclei.

### 2.7. ROS Assay

Renal cryosections were stained with DHE (S0063, Beyotime, Wuhan, China) to detect superoxide, and HK-2 cells were labeled with DCFH-DA (S0033S, Beyotime) to measure intracellular ROS, following manufacturer instructions. Samples were incubated with the respective probes (dark, 37 °C, 30 min), washed with PBS, and imaged on an Olympus IX51 fluorescence microscope [26]. Mean fluorescence intensity was quantified using ImageJ v1.54m (ImageJ, NIH, USA) from ≥5 random fields per sample, using uniform exposure settings and background subtraction.

### 2.8. Western Blot

Proteins from mouse renal and HK-2 cells were lysed on ice in RIPA buffer (BL509A, Biosharp, Shanghai, China), clarified by centrifugation, and quantified by BCA. Equal protein inputs were resolved on SDS–PAGE and electrotransferred to methanol-activated PVDF membranes. Blots were incubated with the primary antibodies (listed in Table S1) at 4 °C overnight and washed. HRP-conjugated secondary antibodies were applied for 90 min. Chemiluminescence was developed using ECL reagent (WBKLS, Millipore, Burlington, MA, USA) and captured within the linear range. Densitometry was performed in ImageJ v1.54m (ImageJ, NIH, USA) with local background subtraction and normalization to housekeeping controls.

### 2.9. Mitochondrial Membrane Potential (MMP) Assay

Using the JC-1 kit (C2006, Beyotime), HK-2 cells were stained in the dark, washed with the kit buffer, and imaged on an Olympus IX51. MMP was determined as the red-to-green fluorescence ratio from randomly chosen fields, quantified in ImageJ with uniform acquisition parameters.

### 2.10. Malondialdehyde (MDA), Glutathione (GSH) ASSAY, Fe^2+^ Levels

Renal tissue homogenates and HK-2 cell lysates were prepared and analyzed using commercial kits (Beyotime, Shanghai, China) according to the manufacturer’s instructions. MDA was measured using the Beyotime Lipid Peroxidation (MDA) Assay Kit (Cat. S0131S), with absorbance read at 532 nm; and GSH was quantified using the Beyotime Total Glutathione Assay Kit (Cat. S0052), read at 412 nm [27]. Values were normalized to protein concentration and expressed relative to controls.

Renal tissue homogenates and HK-2 cell lysates were prepared using BeyoLysis™ Buffer H for Metabolic Assay (Beyotime, Cat. S1066S) according to the manufacturer’s instructions. Samples were acid-treated (5 μL 1 M HCl per 100 μL sample), incubated at 60 °C for 30 min, and centrifuged at 12,000× *g* for 10 min. Supernatants were analyzed for ferrous ion (Fe^2+^) using the Beyotime Ferric and Ferrous Ion Assay Kit (Cat. S1066S) at 590 nm. Fe^2+^ levels were calculated from a standard curve and normalized to protein concentration measured by the BCA Protein Assay Kit (Beyotime, P0012) [27].

### 2.11. Immunofluorescence Staining

After fixation, permeabilization, and blocking, HK-2 cells were incubated with anti- GPX4 (1:100, Cell Signaling, #52455), anti-ACSL4 (1:100, Cell Signaling, # 38493), and anti-Sirt3 (1:200, Cell Signaling, #2627S) at 4 °C overnight. After washing, cells were treated with corresponding fluorescent secondary antibodies. Representative images were captured from random fields using fluorescence microscopy (Olympus IX51).

### 2.12. Statistical Analysis

All statistical analyses were conducted with GraphPad Prism (version 8). Data are summarized as the mean with the standard error of the mean (SEM). The study incorporated random allocation to achieve comparable group sizes, and outcome assessment and data analysis were performed under blinded conditions. No observations were removed from the dataset. For experiments involving three or more groups, we applied one-way analysis of variance (ANOVA) with Tukey’s post hoc procedure for pairwise contrasts. When only two groups were compared, we used Student’s *t* test. A *p* value < 0.05 was considered significant.

## 3. Results

### 3.1. Mul Played a Protective Role in Renal IRI in Mice

**Figure 1:**
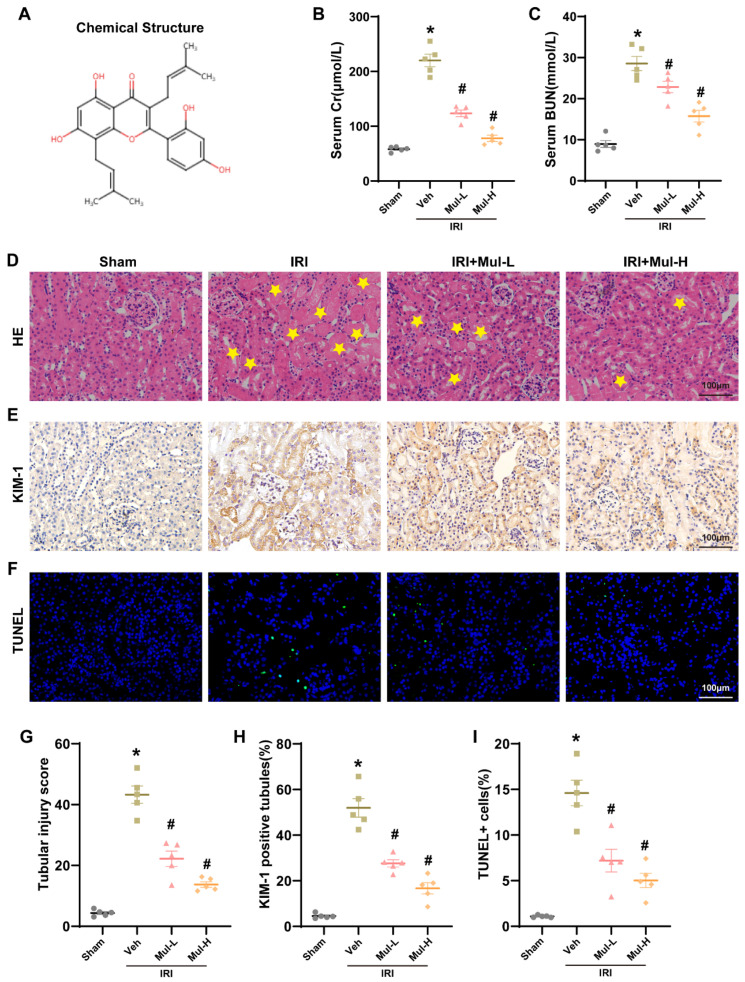
Mul protects the kidneys from IRI. (**A**) The chemical structure of Mul. (**B**) The serum Cr concentrations across various groups. (**C**) The serum BUN levels in different groups. (**D**,**G**) H&E staining of mouse renal tissue with quantitative scoring. Yellow stars in the H&E panels indicate areas of tubular injury. (**E**,**H**) KIM-1 immunohistochemistry with corresponding quantification. (**F**,**I**) TUNEL staining indicating apoptotic cells with accompanying analysis. Bar = 100 μm. Values are expressed as the mean ± SEM. N = 5. * *p* < 0.05, relative to sham group; # *p* < 0.05, relative to IRI group.

We initially established a renal IRI model in mice to evaluate the in vivo protection of Mul. Prior to these experiments, we established the in vivo dosing regimen for Mul. As shown in Figure S1, Mul at 40 mg/kg for three consecutive days achieved the optimal therapeutic effect. Figure 1A shows the chemical structure of Mul and administration of Mul significantly improved the renal function after IRI, with a higher dose (40 mg/kg) having a more significant rescue effect compared with a lower dose (20 mg/kg) of Mul treatment (Figure 1B,C). Histological analysis further revealed that Mul markedly alleviated renal tubular damage induced by IRI (Figure 1D,G). Immunohistochemistry showed increased expression of KIM-1, a sensitive marker of tubular injury, in the IRI group, while Mul pretreatment significantly reduced KIM-1 levels (Figure 1E,H). Moreover, TUNEL staining indicated that Mul effectively attenuated IRI-induced apoptosis in renal tissues (Figure 1F,I). These findings demonstrated that Mul protected against renal IRI in vivo.

### 3.2. Mul Alleviated Oxidative Stress and Ferroptosis Induced by Renal IRI

**Figure 2:**
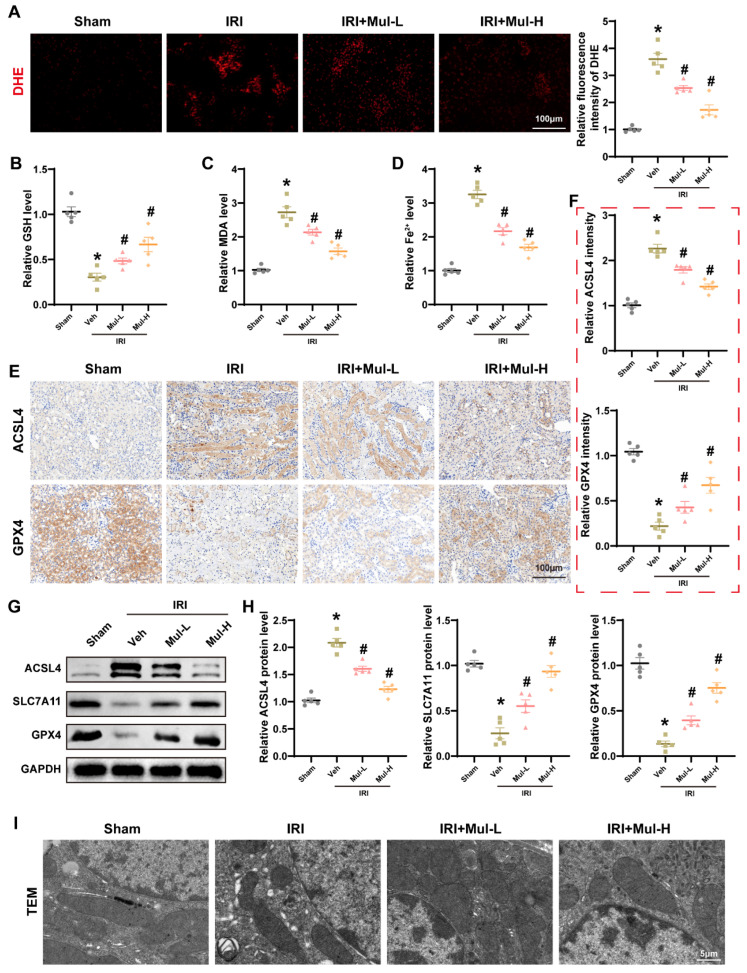
Mul confers renoprotection by limiting oxidative injury and ferroptosis in renal IRI. (**A**) Representative images of DHE staining in mice renal tissues (left) and quantitative analysis (right). Bar = 100 μm. (**B**–**D**) Quantitative analysis of GSH, MDA, and Fe^2+^ levels in the renal of mice receiving different treatments. (**E**,**F**) IHC showing the expression levels and statistical results of GPX4 and ACSL4 in mouse renal under different treatment conditions (left) and quantitative analysis (right). Bar = 100 μm. (**G**,**H**) Western blot showing the expression levels of GPX4, SLC7A11 and ACSL4 in the renal of mice under different treatment conditions and statistical results. (**I**) Representative images of TEM in mice renal tissues. Bar = 5 μm. Values are expressed as the mean ± SEM. N = 5. * *p* < 0.05, relative to sham group; # *p* < 0.05, relative to IRI group.

Recognizing oxidative injury and ferroptosis as central mediators of renal IRI, we examined whether Mul attenuates these mechanisms. IRI caused renal ROS accumulation in renal tissues, whereas Mul treatment was effective in reversing this alteration (Figure 2A). Moreover, the reduction of GSH, accumulation of MDA and accumulation of Fe^2+^ reduced by IRI were also effectively reversed by Mul treatment (Figure 2B–D). In addition, Mul treatment effectively reversed IRI-induced alterations in ferroptosis-related proteins. Protein levels were determined in renal tissues by IHC and Western blot (Figure 2E–H). TEM revealed IRI-induced mitochondrial damage, while Mul treatment preserved mitochondrial ultrastructure (Figure 2I). Together, these results demonstrated Mul concurrently mitigated oxidative stress and ferroptosis in renal IRI.

### 3.3. Mul Attenuated Oxidative Stress to Protect HK-2 Cells from H/R Injury

**Figure 3:**
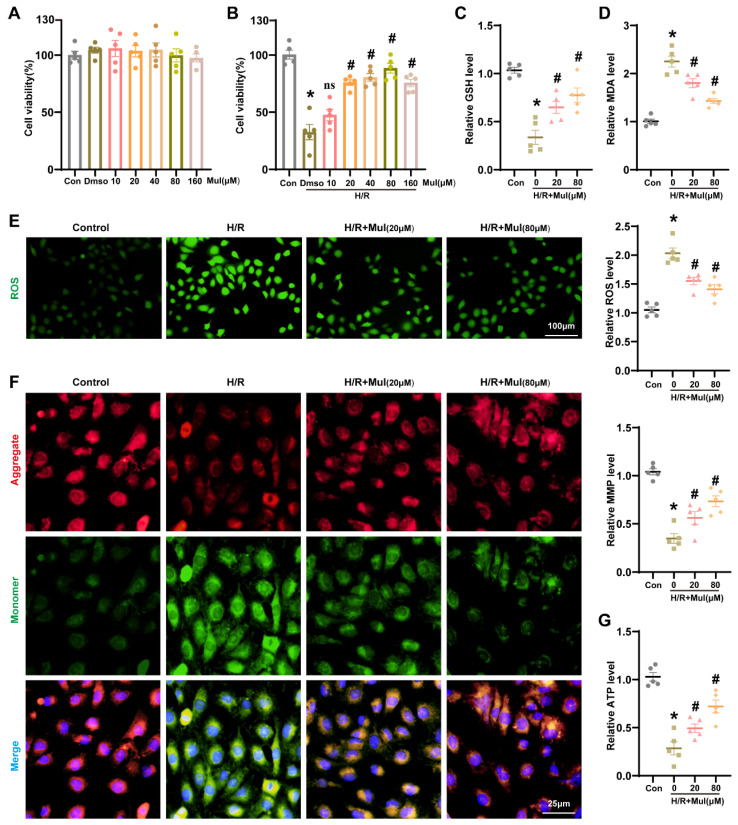
Effects of Mul on oxidative stress during renal H/R injury in HK-2 cells. (**A**,**B**) CCK-8 assay showing cell survival under different treatments. (**C**,**D**) The GSH and MDA levels in HK-2 cells under different treatment conditions. (**E**) Representative images and statistical results of ROS staining in HK-2 cells under different treatment conditions (left) and quantitative analysis (right). Bars = 100 μm. (**F**) Mitochondrial membrane potential was detected by JC-1 assay (left) and related quantitative analysis (right). Bars = 25 μm. (**G**) The ATP content in HK-2 cells under different treatment conditions. Values are expressed as the mean ± SEM. N = 5. * *p* < 0.05, relative to control group; ns *p* > 0.05 and # *p* < 0.05, relative to H/R group.

To thoroughly investigate the protective role of Mul in vitro, HK-2 cells were subjected to an H/R protocol. In parallel normoxic controls, treatment with Mul showed no change in cell viability (Figure 3A). However, following H/R injury, Mul pretreatment significantly improved cell survival in a concentration-dependent manner, with 80 μM showing optimal protective efficacy (Figure 3B). Therefore, the lowest dose of 20 μM and the optimal dose of 80 μM were selected for further exploration in subsequent experiments. Subsequent oxidative stress assays revealed that Mul treatment increased GSH and decreased MDA, effectively reversing H/R-induced alterations (Figure 3C,D). Moreover, ROS staining confirmed that Mul treatment attenuated the H/R-induced ROS elevation (Figure 3E). Because mitochondrial dysfunction closely tracks oxidative injury, we next profiled bioenergetic status. H/R treatment disrupted the MMP and reduced ATP levels, whereas Mul treatment partially restored mitochondrial function (Figure 3F,G). These results confirmed the antioxidant and cytoprotective effects of Mul in vitro.

### 3.4. Mul Suppressed H/R-Induced Ferroptosis in HK-2 Cells

**Figure 4:**
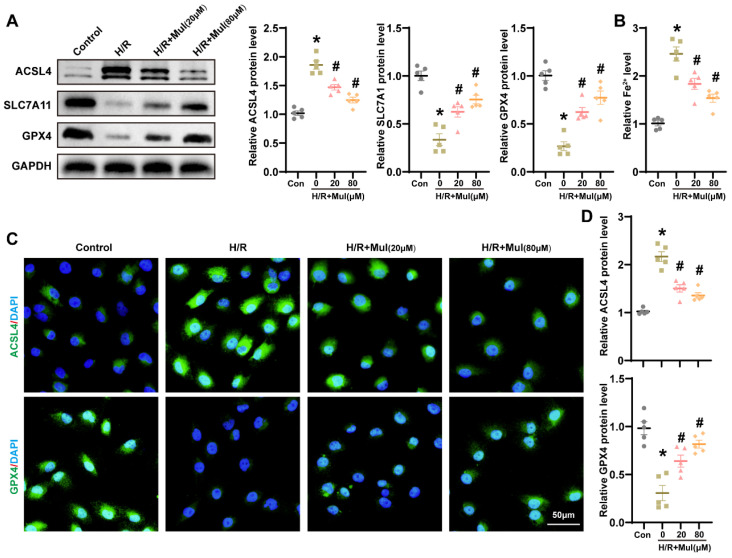
Effects of Mul on ferroptosis during renal H/R injury in HK-2 cells. (**A**) Western blot showing the expression levels of GPX4, SLC7A11 and ACSL4 in HK-2 cells under different treatment conditions and statistical results. (**B**) Quantitative analysis of Fe^2+^ levels in the renal of mice receiving different treatments. (**C**,**D**) Immunofluorescence showing the expression levels of GPX4 and ACSL4 in HK-2 cells under different treatment conditions (**C**) and quantitative analysis (**D**). Bars = 50 μm. Values are expressed as the mean ± SEM. N = 5. * *p* < 0.05, relative to control group; # *p* < 0.05, relative to H/R group.

In order to define Mul’s regulation of ferroptosis in HK-2 cells, we applied an H/R approach in HK-2 cultures and analyzed conventional markers at the protein and functional levels. In agreement with in vivo observations, H/R markedly increased ACSL4 expression and decreased SLC7A11 and GPX4 levels in HK-2 cells, which were reversed by Mul treatment (Figure 4A). Moreover, Mul significantly tempered the elevation of intracellular Fe^2+^ induced by H/R (Figure 4B). Immunofluorescence imaging indicated that Mul treatment led to a lower ACSL4 signal and a higher GPX4 presence, consistent with anti-ferroptosis activity (Figure 4C,D). To determine whether ferroptosis mediates Mul’s benefit in renal IRI, we combined Mul with Fer-1 or with RSL3 to inhibit or promote ferroptosis signaling, respectively. Relative to Mul monotherapy, concurrent exposure to RSL3 abrogated its suppression of ferroptosis signaling. By contrast, co-administration with ferrostatin-1 yielded maximal efficacy (Figure S2). The results collectively demonstrated that Mul counteracted H/R-induced ferroptosis in HK-2 cells.

### 3.5. Mul Enhances Sirt3 Expression Following IRI and H/R

**Figure 5:**
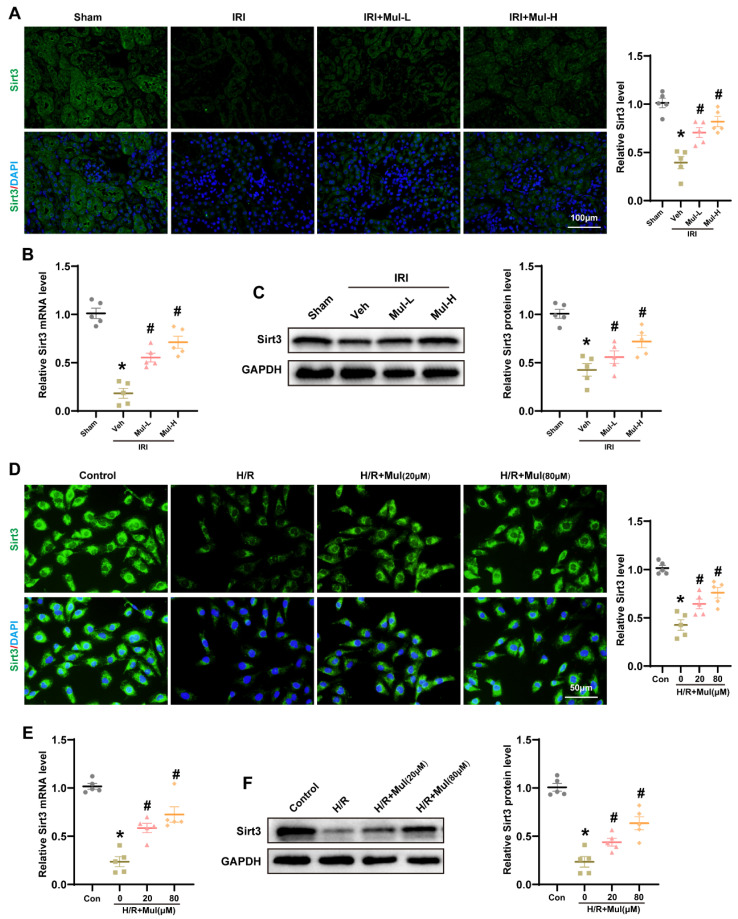
Mul significantly upregulated the expression level of Sirt3 after IRI and H/R. (**A**) Representative image of immunohistochemical staining of mouse renal tissue for Sirt3 (left) and quantitative analysis (right). Bars = 100 μm. (**B**) RT-qPCR detection of Sirt3 mRNA levels in mice renal tissues. (**C**) Western blot detection of Sirt3 protein levels in mice renal tissues (left) and statistical results (right). (**D**) Immunofluorescence showing the expression levels of Sirt3 in HK-2 cells (left) and quantitative analysis (right). Bars = 50 μm. (**E**) RT-qPCR detection of Sirt3 mRNA levels in HK-2 cells. (**F**) Western blot detection of Sirt3 protein levels in HK-2 cells and statistical results. Values are expressed as the mean ± SEM. N = 5. * *p* < 0.05, relative to sham or control group; # *p* < 0.05, relative to IRI or H/R group.

Seeking mechanistic insight, we evaluated the hypothesis that Mul’s protective actions are transmitted through the Sirt3 signaling axis. First, IHC staining revealed decreased Sirt3 expression in renal tissues after IRI, whereas Mul treatment partially restored its expression (Figure 5A). These findings were corroborated by RT-qPCR and Western blot analysis (Figure 5B,C). Similarly, IF staining revealed a pronounced decrease in SIRT3 labeling after H/R in HK-2 cells, while Mul treatment significantly enhanced it (Figure 5D). Consistent results were obtained by RT-qPCR and Western blot analyses of Sirt3 (Figure 5E,F). In summary, these findings endorse a model suggesting that Mul’s cytoprotective actions are facilitated by the regulation of Sirt3.

### 3.6. The Reduction of SIRT3 Hindered Mul’s Ability to Combat Oxidative Stress Triggered by H/R

**Figure 6:**
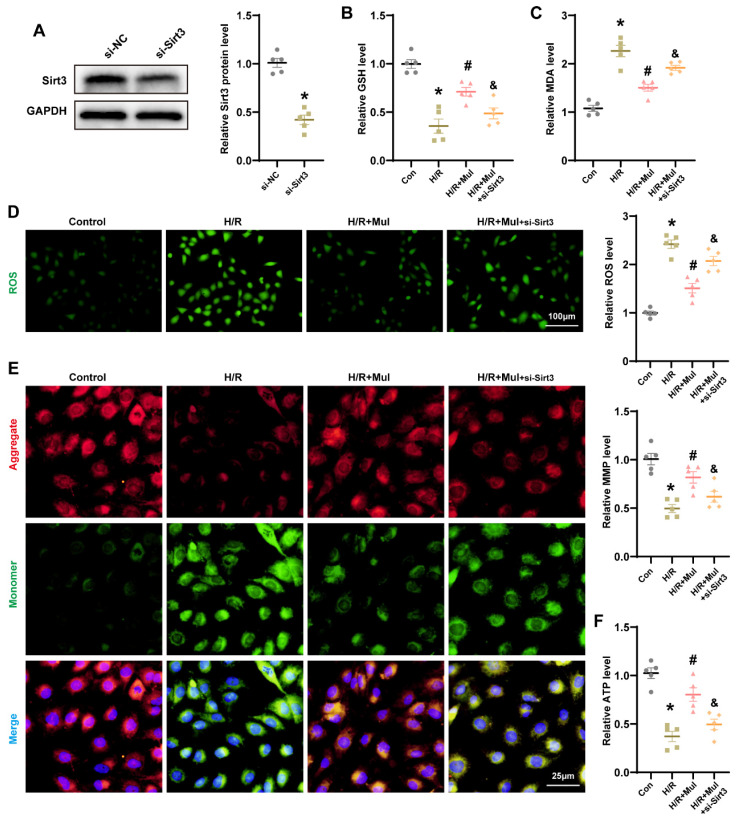
Knockdown of Sirt3 gene blocks Mul resistance to oxidative stress in HK-2 cells. (**A**) Validation of siRNA knockdown efficiency. (**B**,**C**) The GSH and MDA levels in HK-2 cells under different treatment conditions. (**D**) Representative images and statistical results of ROS staining in HK-2 cells under different treatment conditions. Bars = 100 μm. (**E**) Mitochondrial membrane potential was detected by JC-1 assay (left) and related quantitative analysis (right). Bars = 25 μm. (**F**) The ATP content in HK-2 cells under different treatment conditions. Values are expressed as the mean ± SEM. N = 5. * *p* < 0.05, relative to control group; # *p* < 0.05, relative to H/R group; & *p* < 0.05, relative to H/R + Mul group.

To determine whether SIRT3 mediates Mul-induced cytoprotection, we silenced Sirt3 expression in HK-2 cells using siRNA (Figure 6A). Sirt3 silencing eliminated Mul’s protective effects, as indicated by decreased GSH and increased MDA (Figure 6B,C). ROS imaging revealed diminished antioxidant capacity of Mul in the absence of Sirt3 (Figure 6D). To assess mitochondrial performance, we quantified MMP and ATP content. H/R caused significant declines in both indices, consistent with organellar dysfunction. Mul treatment reinstated MMP and replenished ATP, but Sirt3 depletion abrogated these benefits (Figure 6E,F). These findings confirmed that Sirt3 was essential for the antioxidant effects of Mul.

### 3.7. Loss of Sirt3 Blocked Mul’s Anti-Ferroptotic Action During H/R Challenge

**Figure 7:**
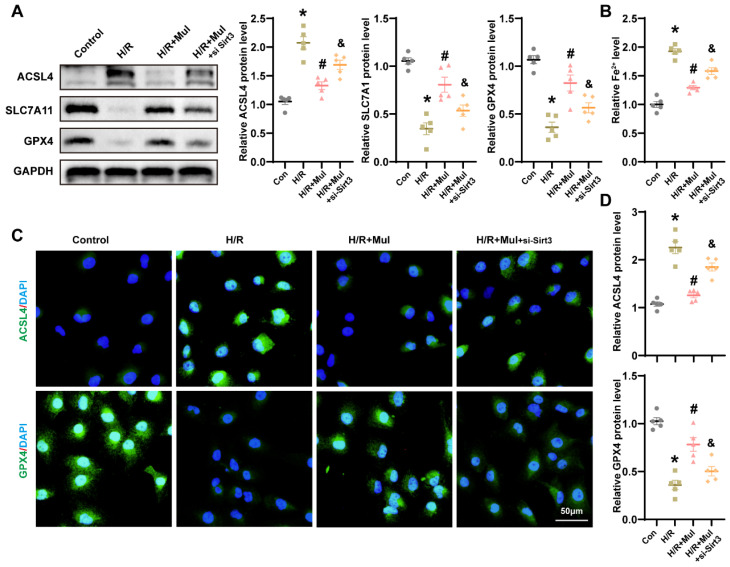
Knockdown of Sirt3 gene blocks Mul resistance to ferroptosis in HK-2 cells. (**A**) Western blot showing the expression levels of GPX4, SLC7A11 and ACSL4 in HK-2 cells under different treatment conditions and statistical results. (**B**) Quantitative analysis of Fe^2+^ levels in the renal of mice receiving different treatments. (**C**,**D**) Immunofluorescence showing the expression levels of GPX4 and ACSL4 in HK-2 cells under different treatment conditions (**C**) and quantitative analysis (**D**). Bars = 50 μm. Values are expressed as the mean ± SEM. N = 5. * *p* < 0.05, relative to control group; # *p* < 0.05, relative to H/R group; & *p* < 0.05, relative to H/R + Mul group.

We further examined whether Sirt3 knockdown affects Mul-mediated ferroptosis suppression under H/R conditions. Western blot analysis revealed that Mul treatment downregulated ACSL4 and upregulated SLC7A11 and GPX4. However, Sirt3 silencing abolished these effects, reversing Mul’s modulation of ACSL4, SLC7A11, and GPX4 (Figure 7A). Consistent with this, Mul reduced H/R-induced Fe^2+^ accumulation, but this protective effect was lost upon Sirt3 depletion (Figure 7B). IF staining further confirmed that Sirt3 knockdown blocked Mul’s regulatory effects on ACSL4 and GPX4 expression during H/R (Figure 7C,D). Collectively, these findings indicate that Mul mitigates ferroptosis in renal IRI via a Sirt3-dependent mechanism.

### 3.8. Sirt3 Depletion Negated Mul’s Protection from IRI-Driven Oxidative Injury and Ferroptosis

**Figure 8:**
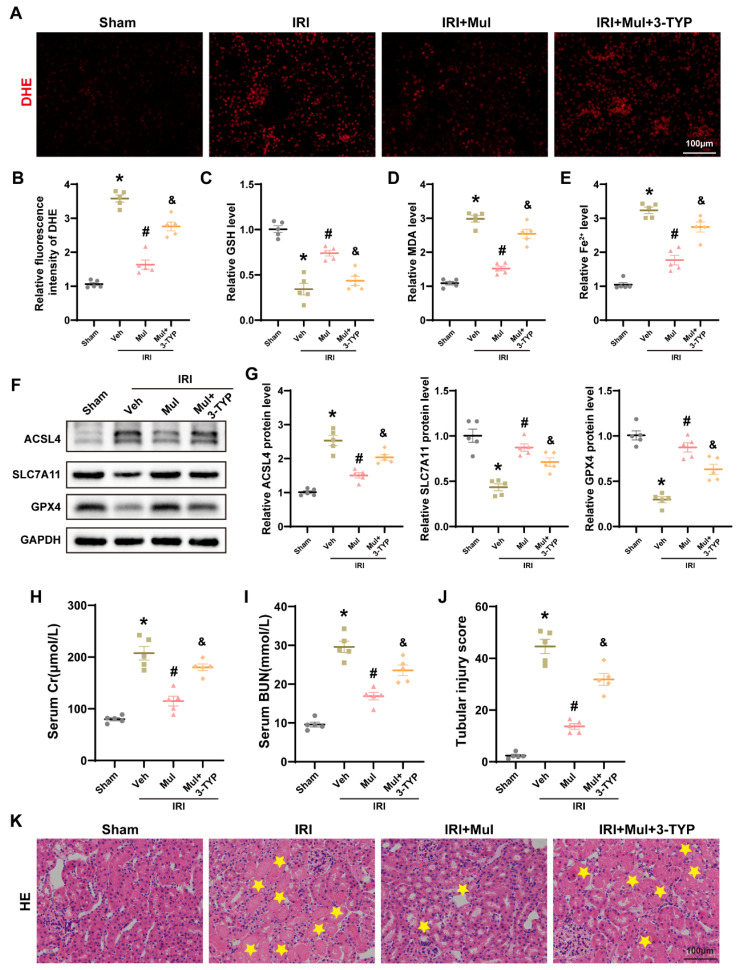
Sirt3 knockdown suppressed the protective effects of Mul against IRI-induced oxidative stress and ferroptosis. (**A**,**B**) Representative images of DHE staining in mice renal tissues (**A**) and quantitative analysis (**B**). Bar = 100 μm. (**C**–**E**) Quantitative analysis of GSH, MDA, and Fe^2+^ levels in the renal of mice receiving different treatments. (**F**,**G**) Western blot showing the expression levels of GPX4, SLC7A11 and ACSL4 in the renal of mice under different treatment conditions and statistical results. (**H**) The serum Cr levels in different groups. (**I**) The serum BUN levels in different groups. (**J**,**K**) Representative image of HE staining of mice renal tissue (**K**) and associated quantitative analysis (**J**). Yellow stars in the H&E panels indicate areas of tubular injury. Bar = 100 μm. Values are expressed as the mean ± SEM. N = 5. * *p* < 0.05, relative to Sham group; # *p* < 0.05, relative to IRI group; & *p* < 0.05, relative to IRI + Mul group.

To verify the dependence of Mul on Sirt3 in vivo, we employed pharmacologic blockade in vivo by giving 3-TYP to mice undergoing renal IRI. Compared with Mul treatment alone, the combination of Mul and 3-TYP resulted in increased ROS levels, as detected by immunofluorescence (Figure 8A,B). In addition, 3-TYP reduced the levels of GSH, GPX4, and SLC7A11, while enhancing ACSL4 expression, MDA accumulation, and Fe^2+^ deposition (Figure 8C–G). Histological analysis further showed that, relative to Mul treatment alone, the Mul+3-TYP group exhibited higher serum Cr and BUN levels, with aggravated tubular injury (Figure 8H–K). Overall, these results suggest that Mul’s renal-protective effect relies on Sirt3.

## 4. Discussion

**Figure 9:**
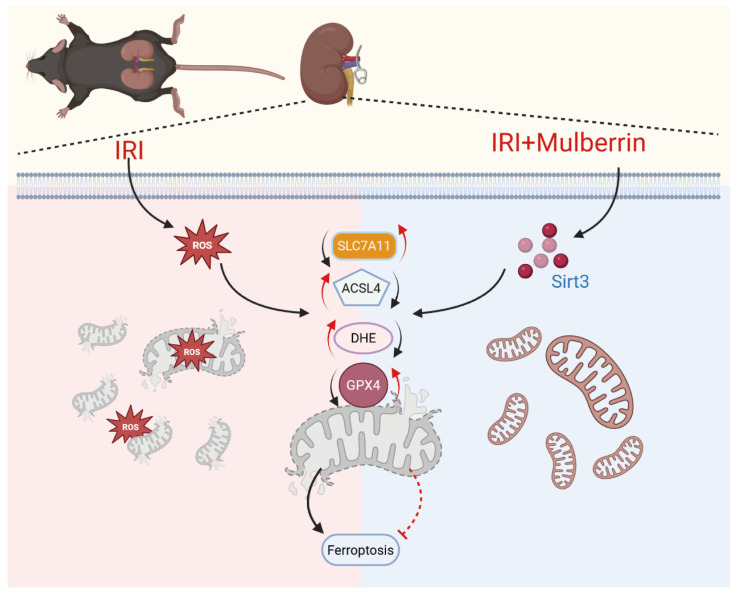
Mulberrin alleviates renal ischemia–reperfusion by inhibiting ferroptosis and oxidative stress through the Sirt3 activation.

Using murine IRI and HK-2 H/R paradigms, we found that Mul markedly reduces renal IRI, providing, to the best of our knowledge, the inaugural evidence of its efficacy across complementary experimental systems. Mechanistically, Mul alleviated oxidative stress and inhibited ferroptosis through activation of Sirt3. Silencing of Sirt3 abolished the renoprotective effects of Mul, establishing Sirt3 as a critical node for its antioxidant and anti-ferroptosis functions (Figure 9).

IRI is an established pathogenic factor in AKI [3]. Despite ongoing development of therapeutic agents, clinical translation remains limited by adverse effects and drug resistance [17,28]. Consequently, identifying natural compounds with renoprotective properties has emerged as a critical research focus. Previous studies have demonstrated that Mul exerts diverse pharmacological activities, particularly anti-inflammatory and antioxidant effects, which are linked to activation of Nrf2/HO-1 signaling and to the dampening of oxidative injury and apoptosis [23]. Mul has shown protective roles in conditions including spinal cord injury, Parkinson’s disease, and hepatic fibrosis [22,23,29]. Similarly to these studies, we observed that Mul attenuated renal pathological injury caused by ischemia–reperfusion and reduced Cr as well as BUN levels.

Accumulating evidence highlights ferroptosis as a critical driver of renal IRI, as excessive iron accumulation, lipid peroxidation, and depletion of GPX4 aggravate tubular cell death and renal dysfunction [9,30]. Previous studies have shown that chelation of iron ions, inhibition of ACSL4 expression, and upregulation of GPX4 levels could ameliorate renal IRI by inhibiting ferroptosis [31,32,33]. Aligning with earlier observations, our experimental data also showed that IRI and H/R promoted ferroptosis hallmarks, including Fe^2+^ overload, MDA accumulation, decreased GSH levels, and disturbed expression of key regulators such as ACSL4, GPX4, and SLC7A11. Administration of Mul restored the altered indices in a dose-responsive fashion, implying inhibition of ferroptosis and stabilization of cellular redox homeostasis under ischemic conditions. A range of plant-derived flavonoids, exemplified by quercetin and curcumin, exhibit protective activity in renal IRI and additional acute injury settings through coordinated inhibition of ferroptosis and attenuation of oxidative stress. These compounds reduce lipid peroxidation, restore glutathione levels, and activate antioxidant pathways such as Nrf2/HO-1, thereby preserving tubular cell integrity [34,35]. Our findings extend previous reports that flavonoids act as ferroptosis inhibitors and provide new insight into Mul’s therapeutic potential. Compared with quercetin and curcumin, Mul is a prenylated flavonoid derived from Morus alba whose prenyl group may alter membrane affinity and bioactivity. Prenylation can therefore change both target engagement and pharmacokinetic properties relative to non-prenylated flavonoids [36].

Importantly, our study identified Sirt3 as a central mediator of Mul’s renoprotective effects. Sirt3 is a mitochondrial NAD^+^-dependent deacetylase known to preserve mitochondrial integrity, reduce ROS production, and regulate ferroptosis through stabilization of GPX4 and modulation of iron metabolism [15]. Previous studies have reported that Sirt3 expression is markedly decreased in various models of renal injury [17,37,38]. Mitochondrial dysfunction is now recognized as a central hallmark of ferroptosis, during which excessive mitochondrial oxidative stress promotes cell death [39,40]. Functionally, Sirt3 maintains mitochondrial activity and redox equilibrium through the deacetylation of key antioxidant enzymes [38,41]. Across multiple models, activation of Sirt3 has been associated with inhibition of ferroptosis, corresponding to improved outcomes in diabetic nephropathy, reduced doxorubicin cardiotoxicity, and attenuation of femoral head osteonecrosis [42,43,44]. Echoing earlier work linking Sirt3 to kidney defense, our results showed that Mul elevated Sirt3 expression and partially normalized MMP and ATP synthesis in H/R-injured HK-2 cells. Notably, Sirt3 knockdown abolished Mul-mediated antioxidant and anti-ferroptosis effects, confirming the requirement of Sirt3 in this process. These findings suggest that Mul enhances mitochondrial resilience and ferroptosis resistance through a Sirt3-dependent mechanism.

From a translational medicine perspective, Mul exhibits considerable clinical potential, particularly in the prevention and treatment of AKI caused by IRI, such as that occurring during renal transplantation. Our study evaluated the preventive renoprotective effects of Mul and preliminarily elucidated its underlying mechanisms. Since post-ischemic therapeutic data are critical for clinical translation, future studies should further investigate the protective efficacy of Mul when administered after ischemic injury. Although we identified Sirt3 as a critical mediator, the precise downstream molecular targets through which Sirt3 regulates ferroptosis in renal IRI remain to be clarified. In particular, whether Mul treatment or Sirt3 activation induces deacetylation of key ferroptosis-related proteins (such as GPX4 or mitochondrial electron transport components) has not been fully investigated. Moreover, comprehensive pharmacokinetic and pharmacodynamic analyses of Mul are lacking, which limits the understanding of its in vivo mechanisms of action. Therefore, future studies should focus on elucidating the downstream regulatory pathways of Sirt3 and conducting detailed pharmacological evaluations of Mul in renal IRI.

## 5. Conclusions

In conclusion, our findings demonstrated that Mul protected against renal IRI by activating Sirt3 and suppressing ferroptosis. This work highlighted Mul as a novel therapeutic candidate for AKI and provided a mechanistic rationale for targeting the Sirt3–ferroptosis axis in renal protection.

## Data Availability

The original contributions presented in this study are included in the article/Supplementary Materials. Further inquiries can be directed to the corresponding author.

## Supplementary Materials

The following supporting information can be downloaded at https://www.mdpi.com/article/10.3390/biomedicines13112687/s1. Table S1: Antibodies for Western blotting; Figure S1: (A,B) Renal function was evaluated after treatment with different doses of Lut by measuring Cr and BUN levels. (C,D) Renal function was evaluated after treatment with Lut (80 mg/kg) for different durations by measuring Cr and BUN levels. Values are expressed as the mean ± SEM. N = 5. * *p* < 0.05, relative to sham group; ns *p* > 0.05, # *p* < 0.05, relative to IRI group; Figure S2: (A) Representative images and statistical results of ROS staining in HK-2 cells under different treatment conditions (left) and quantitative analysis (right). Bars = 100 μm. (B–E) Quantitative analysis of GSH, MDA, ATP and Fe^2+^ levels in HK-2 cells after different treatments. (F,G) WB detection of ACSL4, SLC7A11 and GPX4 protein levels in HK-2 cells (F) and related quantitative analysis (G). Values are expressed as the mean ± SEM. N = 5. * *p* < 0.05, relative to IRI group; # *p* < 0.05, relative to IRI+Mul group.

## References

[1] Ostermann M., Lumlertgul N., Jeong R., See E., Joannidis M., James M. (2025). Acute kidney injury. Lancet.

[2] Matsuura R., Doi K., Rabb H. (2023). Acute kidney injury and distant organ dysfunction-network system analysis. Kidney Int..

[3] Funk J.A., Schnellmann R.G. (2012). Persistent disruption of mitochondrial homeostasis after acute kidney injury. Am. J. Physiol. Renal Physiol..

[4] Bhargava P., Schnellmann R.G. (2017). Mitochondrial energetics in the kidney. Nat. Rev. Nephrol..

[5] Yu H., Jin F., Liu D., Shu G., Wang X., Qi J., Sun M., Yang P., Jiang S., Ying X. (2020). ROS-responsive nano-drug delivery system combining mitochondria-targeting ceria nanoparticles with atorvastatin for acute kidney injury. Theranostics.

[6] Dixon S.J., Lemberg K.M., Lamprecht M.R., Skouta R., Zaitsev E.M., Gleason C.E., Patel D.N., Bauer A.J., Cantley A.M., Yang W.S. (2012). Ferroptosis: An iron-dependent form of nonapoptotic cell death. Cell.

[7] Zhou L., Han S., Guo J., Qiu T., Zhou J., Shen L. (2022). Ferroptosis-A New Dawn in the Treatment of Organ Ischemia-Reperfusion Injury. Cells.

[8] Shi L., Song Z., Li Y., Huang J., Zhao F., Luo Y., Wang J., Deng F., Shadekejiang H., Zhang M. (2023). MiR-20a-5p alleviates kidney ischemia/reperfusion injury by targeting ACSL4-dependent ferroptosis. Am. J. Transplant..

[9] Deng Y., Zeng L., Liu H., Zuo A., Zhou J., Yang Y., You Y., Zhou X., Peng B., Lu H. (2024). Silibinin attenuates ferroptosis in acute kidney injury by targeting FTH1. Redox Biol..

[10] Hosohata K., Harnsirikarn T., Chokesuwattanaskul S. (2022). Ferroptosis: A Potential Therapeutic Target in Acute Kidney Injury. Int. J. Mol. Sci..

[11] Shi Z., Du Y., Zheng J., Tang W., Liang Q., Zheng Z., Liu B., Sun H., Wang K., Shao C. (2024). Liproxstatin-1 Alleviated Ischemia/Reperfusion-Induced Acute Kidney Injury via Inhibiting Ferroptosis. Antioxidants.

[12] Zhang J., Ren D., Fedorova J., He Z., Li J. (2020). SIRT1/SIRT3 Modulates Redox Homeostasis during Ischemia/Reperfusion in the Aging Heart. Antioxidants.

[13] Klimova N., Fearnow A., Long A., Kristian T. (2020). NAD(+) precursor modulates post-ischemic mitochondrial fragmentation and reactive oxygen species generation via SIRT3 dependent mechanisms. Exp. Neurol..

[14] Li N., Xiong R., Li G., Wang B., Geng Q. (2023). PM2.5 contributed to pulmonary epithelial senescence and ferroptosis by regulating USP3-SIRT3-P53 axis. Free Radic. Biol. Med..

[15] Zeng L., Hu P., Wang X., Ding X., Wang Q., Luo L., Zhang Y., Li M., Zhao Y., Li S. (2025). Sirtuin-3 activation by honokiol attenuated anesthesia/surgery-induced cognitive impairment and neuronal ferroptosis via inhibiting mitochondrial GPX4 acetylation. J. Nanobiotechnol..

[16] Ma D., Fang W., Cai L., Li W., Su H. (2025). Norepinephrine exacerbates LPS-induced cardiomyopathy via SIRT3/HO-1 axis-mediated ferroptosis. Crit. Care.

[17] Xia K., Jin Z., Qiu Q., Zhou Y., Lu Y., Qiu T., Zhou J., Chen Z. (2024). Ligustilide alleviates oxidative stress during renal ischemia-reperfusion injury through maintaining Sirt3-dependent mitochondrial homeostasis. Phytomedicine.

[18] Liu D., Tang S., Gan L., Cui W. (2021). Renal-Protective Effects and Potential Mechanisms of Traditional Chinese Medicine after Ischemia-Reperfusion Injury. Evid. Based Complement. Altern. Med..

[19] Li Y., Dong M., Qin H., An G., Cen L., Deng L., Cui H. (2025). Mulberrin suppresses gastric cancer progression and enhances chemosensitivity to oxaliplatin through HSP90AA1/PI3K/AKT axis. Phytomedicine.

[20] Morante-Carriel J., Živković S., Nájera H., Sellés-Marchart S., Martínez-Márquez A., Martínez-Esteso M.J., Obrebska A., Samper-Herrero A., Bru-Martínez R. (2024). Prenylated Flavonoids of the Moraceae Family: A Comprehensive Review of Their Biological Activities. Plants.

[21] Kim Y.S., Kwon E.B., Kim B., Chung H.S., Choi G., Kim Y.H., Choi J.G. (2022). Mulberry Component Kuwanon C Exerts Potent Therapeutic Efficacy In Vitro against COVID-19 by Blocking the SARS-CoV-2 Spike S1 RBD:ACE2 Receptor Interaction. Int. J. Mol. Sci..

[22] Xia P., Gao X., Duan L., Zhang W., Sun Y.F. (2018). Mulberrin (Mul) reduces spinal cord injury (SCI)-induced apoptosis, inflammation and oxidative stress in rats via miroRNA-337 by targeting Nrf-2. Biomed. Pharmacother..

[23] Ge C., Tan J., Lou D., Zhu L., Zhong Z., Dai X., Sun Y., Kuang Q., Zhao J., Wang L. (2022). Mulberrin confers protection against hepatic fibrosis by Trim31/Nrf2 signaling. Redox Biol..

[24] Liu H., Wang L., Weng X., Chen H., Du Y., Diao C., Chen Z., Liu X. (2019). Inhibition of Brd4 alleviates renal ischemia/reperfusion injury-induced apoptosis and endoplasmic reticulum stress by blocking FoxO4-mediated oxidative stress. Redox Biol..

[25] Han S., Guo J., Kong C., Li J., Lin F., Zhu J., Wang T., Chen Q., Liu Y., Hu H. (2024). ANKRD1 aggravates renal ischaemia-reperfusion injury via promoting TRIM25-mediated ubiquitination of ACSL3. Clin. Transl. Med..

[26] Xia K., Guo J., Yu B., Wang T., Qiu Q., Chen Q., Qiu T., Zhou J., Zheng S. (2024). Sentrin-specific protease 1 maintains mitochondrial homeostasis through targeting the deSUMOylation of sirtuin-3 to alleviate oxidative damage induced by hepatic ischemia/reperfusion. Free Radic. Biol. Med..

[27] Su Z., Li P., Ding W., Gao Y. (2024). Urolithin A improves myocardial ischemia-reperfusion injury by attenuating oxidative stress and ferroptosis through Nrf2 pathway. Int. Immunopharmacol..

[28] Xu C., Wang H., Wang H., Man J., Deng Y., Li Y., Cheng K., Niu J., Gui H., Fu S. (2025). Schisandrin B regulates mitochondrial dynamics via AKT1 activation and mitochondrial targeting to ameliorate renal ischemia-reperfusion injury. Phytomedicine.

[29] Cao W., Dong Y., Zhao W., Lu X., Sun L. (2019). Mulberrin attenuates 1-methyl-4-phenyl-1,2,3,6- tetrahydropyridine (MPTP)-induced Parkinson’s disease by promoting Wnt/β-catenin signaling pathway. J. Chem. Neuroanat..

[30] Ma Y., Fei S., Chen X., Gui Y., Zhou B., Xiang T., Liu J., Yue K., Li Q., Jiang W. (2024). Chemerin attenuates acute kidney injury by inhibiting ferroptosis via the AMPK/NRF2/SLC7A11 axis. Commun. Biol..

[31] Gao G., Xia H., Shi J., Zheng P., Wu W., Wu S., Qi T., Song H., Gu Y., Li J. (2025). Carbon Dot Nanozymes with Ferrous Ion-Chelating and Antioxidative Activity Inhibiting Ferroptosis to Alleviate Renal Ischemia-Reperfusion Injury. Small.

[32] Tao W.H., Shan X.S., Zhang J.X., Liu H.Y., Wang B.Y., Wei X., Zhang M., Peng K., Ding J., Xu S.X. (2022). Dexmedetomidine Attenuates Ferroptosis-Mediated Renal Ischemia/Reperfusion Injury and Inflammation by Inhibiting ACSL4 via α2-AR. Front. Pharmacol..

[33] Zhao W., Nikolic-Paterson D.J., Li K., Li Y., Wang Y., Chen X., Duan Z., Zhang Y., Liu P., Lu S. (2024). Selenium binding protein 1 protects renal tubular epithelial cells from ferroptosis by upregulating glutathione peroxidase 4. Chem. Biol. Interact..

[34] Guerrero-Hue M., García-Caballero C., Palomino-Antolín A., Rubio-Navarro A., Vázquez-Carballo C., Herencia C., Martín-Sanchez D., Farré-Alins V., Egea J., Cannata P. (2019). Curcumin reduces renal damage associated with rhabdomyolysis by decreasing ferroptosis-mediated cell death. FASEB J..

[35] Wang Y., Quan F., Cao Q., Lin Y., Yue C., Bi R., Cui X., Yang H., Yang Y., Birnbaumer L. (2021). Quercetin alleviates acute kidney injury by inhibiting ferroptosis. J. Adv. Res..

[36] Lv H.W., Wang Q.L., Luo M., Zhu M.D., Liang H.M., Li W.J., Cai H., Zhou Z.B., Wang H., Tong S.Q. (2023). Phytochemistry and pharmacology of natural prenylated flavonoids. Arch. Pharm. Res..

[37] Deng Z., He M., Hu H., Zhang W., Zhang Y., Ge Y., Ma T., Wu J., Li L., Sun M. (2024). Melatonin attenuates sepsis-induced acute kidney injury by promoting mitophagy through SIRT3-mediated TFAM deacetylation. Autophagy.

[38] Yuan Y., Yuan L., Yang J., Liu F., Liu S., Li L., Liao G., Tang X., Cheng J., Liu J. (2024). Autophagy-deficient macrophages exacerbate cisplatin-induced mitochondrial dysfunction and kidney injury via miR-195a-5p-SIRT3 axis. Nat. Commun..

[39] Li J., Jia Y.C., Ding Y.X., Bai J., Cao F., Li F. (2023). The crosstalk between ferroptosis and mitochondrial dynamic regulatory networks. Int. J. Biol. Sci..

[40] She H., Tan L., Du Y., Zhou Y., Guo N., Zhang J., Du Y., Wang Y., Wu Z., Ma C. (2023). VDAC2 malonylation participates in sepsis-induced myocardial dysfunction via mitochondrial-related ferroptosis. Int. J. Biol. Sci..

[41] Zhang S., Wu X., Wang J., Shi Y., Hu Q., Cui W., Bai H., Zhou J., Du Y., Han L. (2022). Adiponectin/AdiopR1 signaling prevents mitochondrial dysfunction and oxidative injury after traumatic brain injury in a SIRT3 dependent manner. Redox Biol..

[42] Liu X., Li D., Pi W., Wang B., Xu S., Yu L., Yao L., Sun Z., Jiang J., Mi Y. (2022). LCZ696 protects against doxorubicin-induced cardiotoxicity by inhibiting ferroptosis via AKT/SIRT3/SOD2 signaling pathway activation. Int. Immunopharmacol..

[43] Li Q., Liao J., Chen W., Zhang K., Li H., Ma F., Zhang H., Han Q., Guo J., Li Y. (2022). NAC alleviative ferroptosis in diabetic nephropathy via maintaining mitochondrial redox homeostasis through activating SIRT3-SOD2/Gpx4 pathway. Free Radic. Biol. Med..

[44] Fan Y., Chen Z., Wang H., Jiang M., Lu H., Wei Y., Hu Y., Mo L., Liu Y., Zhou C. (2025). Isovitexin targets SIRT3 to prevent steroid-induced osteonecrosis of the femoral head by modulating mitophagy-mediated ferroptosis. Bone Res..

