# Supplementary material for: Mulberrin Alleviates Renal Ischemia–Reperfusion by Inhibiting Ferroptosis and Oxidative Stress Through Sirt3 Activation
Source: Biomedicines. 2025 Oct 31;13(11):2687. doi: 10.3390/biomedicines13112687 (PMC12649862; doi:10.3390/biomedicines13112687)
Supplement: Supplementary file 1 [file biomedicines-13-02687-s001.zip › biomedicines-3875422-supplementary.pdf]

Supplementary Material

Mulberrin Alleviates Renal Ischemia–Reperfusion by Inhibiting Ferroptosis and Oxidative Stress Through Sirt3 Activation

Qiangmin Qiu [1,2,†], Zhan Chen [1,2,†], Wenbin Yang [3], Yujie Zhou [1,2], Nan Jiang [1,2], Jiahao Jiang [1,2], Dalin He [1,2], Yifan Lu [1,2], Bo Yu [1,2], Tao Qiu [1,2,*] and Jiangqiao Zhou [1,2,*]

[1] Department of Organ Transplantation, Renmin Hospital of Wuhan University, Wuhan 430060, China; 2015302180359@whu.edu.cn (Q.Q.); applepure@163.com (Z.C.); zhouyujie0514@163.com (Y.Z.); nanjiang0730@163.com (N.J.); 17607137591@163.com (J.J.); 17326375859@163.com (D.H.); yifanlu26@gmail.com (Y.L.); yubo1995@whu.edu.cn (B.Y.)

[2] Department of Urology, Renmin Hospital of Wuhan University, Wuhan 430060, China

[3] Department of Emergency, Renmin Hospital of Wuhan University, Wuhan 430060, China; 18207135709@163.com

* Correspondence: qiutao@whu.edu.cn (T.Q.); zhoujq@whu.edu.cn (J.Z.); Tel.: +86-13995632367 (T.Q.); +86-18207182905 (J.Z.); Fax: +86-027-88041911 (T.Q. & J.Z.)

† These authors contributed equally to this work.

This file contains a supplementary table and two supplementary figures.

Table S1. Antibodies for Western blotting.

Antibody	Dilution	Source	Cat No.
Rabbit anti-ACSL4	1:1000	Proteintech	22401-1-AP
Rabbit anti-SLC7A11	1:1000	Proteintech	26864-1-AP
Rabbit anti-GPX4	1:1000	CST	#52455
Rabbit anti-Sirt3	1:1000	CST	#2627S
Rabbit anti-GAPDH	1:5000	Abcam	Ab8245

Supplementary figures.

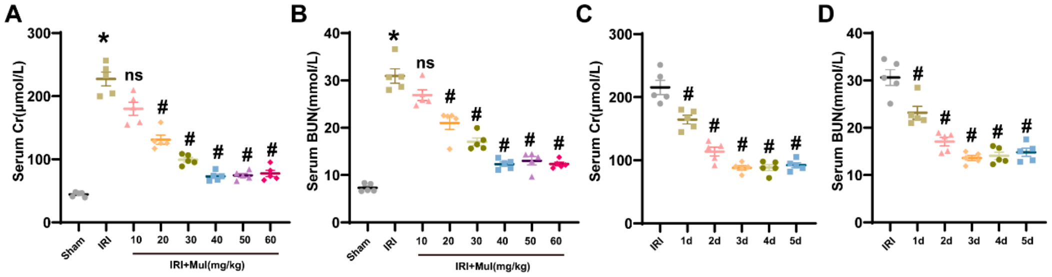

Figure S1. (**A**,**B**) Renal function was evaluated after treatment with different doses of Lut by measuring Cr and BUN levels. (**C**,**D**) Renal function was evaluated after treatment with Lut (80 mg/kg) for different durations by measuring Cr and BUN levelsValues are expressed as the mean ± SEM. N = 5. * $p < 0.05$, relative to sham group; ns $p > 0.05$, # $p < 0.05$, relative to IRI group.

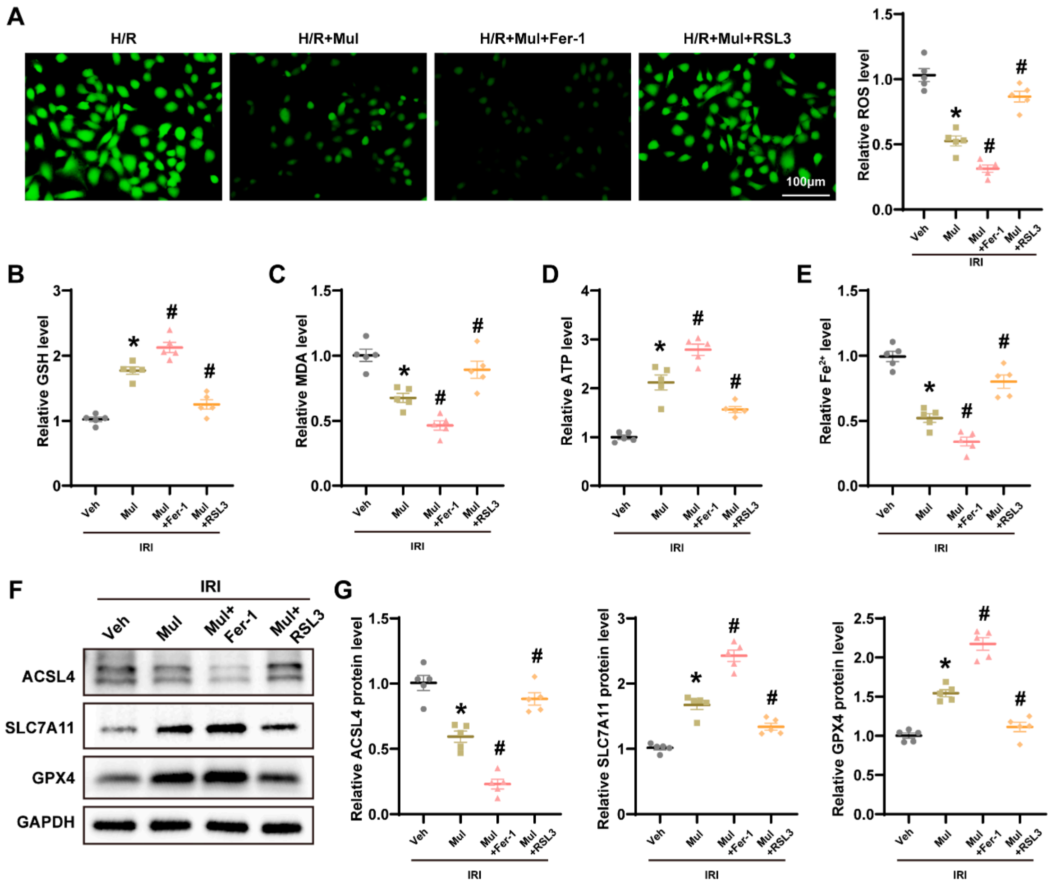

Figure S2. (**A**) Representative images and statistical results of ROS staining in HK-2 cells under different treatment conditions (left) and quantitative analysis (right). Bars = 100 μm. (**B**–**E**) Quantitative analysis of GSH, MDA, ATP and Fe^{2+} levels in HK-2 cells after different treatments. (**F**,**G**) WB detection of ACSL4, SLC7A11 and GPX4 protein levels in HK-2 cells (**F**) and related quantitative analysis (**G**). Values are expressed as the mean ± SEM. N = 5. * $p < 0.05$, relative to IRI group; # $p < 0.05$, relative to IRI+Mul group.
